# Excessive Daytime Sleepiness and Associated Cardiometabolic Factors in Latino Individuals of Mexican Ancestry at High Risk of Type 2 Diabetes: An El Banco Biobank Cross-Sectional Study

**DOI:** 10.3390/nu17152476

**Published:** 2025-07-29

**Authors:** Ludovica Verde, Dawn K. Coletta, Yann C. Klimentidis, Linsday N. Kohler, Lisa Soltani, Oscar D. Parra, Sairam Parthasarathy, Lawrence J. Mandarino, Giovanna Muscogiuri

**Affiliations:** 1 Department of Public Health, University of Naples Federico II, Via Sergio Pansini 5, 80131 Naples, Italy; 2 Division of Endocrinology, Department of Medicine, University of Arizona, Tucson, AZ 85721, USA; 3 Department of Physiology, University of Arizona, Tucson, AZ 85721, USA; 4 Center for Disparities in Diabetes, Obesity and Metabolism, University of Arizona, Tucson, AZ 85721, USA; 5 Department of Epidemiology and Biostatistics, Mel and Enid Zuckerman College of Public Health, Tucson, AZ 85721, USA; 6 Epidemiology Division, Pima County Health Department, Tucson, AZ 85721, USA; 7 Department of Internal Medicine, El Rio Health, Tucson, AZ 85721, USA; 8 University of Arizona Health Sciences Center for Sleep, Circadian & Neuroscience, University of Arizona, Tucson, AZ 85721, USA; 9 Unità di Endocrinologia, Diabetologia e Andrologia, Dipartimento di Medicina Clinica e Chirurgia, University of Naples Federico II, Via Sergio Pansini 5, 80131 Naples, Italy; 10 Cattedra Unesco “Educazione Alla Salute E Allo Sviluppo Sostenibile”, University of Naples Federico II, 80131 Naples, Italy

**Keywords:** sleepiness, HbA1c, type 2 diabetes, diet, Latinos, disparities, cardiometabolic risk factors

## Abstract

**Background/Objectives**: Latinos, particularly those of Mexican ancestry, experience high rates of type 2 diabetes and sleep disturbances, exacerbating adverse health outcomes. This study aimed to examine the prevalence of excessive daytime sleepiness and its associations with diet, cardiometabolic risk factors, and glycemic control in this population. **Methods**: This cross-sectional study utilized data from the El Banco por Salud biobank, including 1685 participants (aged 52.6 ± 14.5 years, BMI: 32.4 ± 7.0 kg/m^2^) recruited from Federally Qualified Community Health Centers. Excessive daytime sleepiness was assessed using the Epworth Sleepiness Scale, while dietary information was obtained via the Brief Dietary Assessment Tool for Hispanics. Primary outcomes included cardiometabolic risk factors and glycated hemoglobin (HbA1c) levels. **Results**: Excessive daytime sleepiness (Epworth Sleepiness Scale > 10) was present in 22.0% of participants and was associated with higher BMI (*p* < 0.001), larger waist circumference (*p* = 0.002), poorer diet quality, increased dyslipidemia (*p* = 0.036), and elevated HbA1c (*p* = 0.007). Linear regression analyses confirmed that excessive daytime sleepiness was significantly associated with higher HbA1c levels, both in unadjusted (R^2^ = 0.011; *p* < 0.001) and adjusted for demographic, anthropometric, and socioeconomic factors (R^2^ = 0.107; *p* = 0.004) models. **Conclusions**: Excessive daytime sleepiness among Latinos of Mexican ancestry is associated with unhealthy dietary patterns and poor glycemic control, highlighting the need for targeted interventions addressing sleep and dietary habits in this vulnerable population.

## 1. Introduction

Emerging evidence has assigned an important role to sleep as a modulator of metabolic homeostasis [1]. The impact of sleep disturbances on cardiometabolic function encompasses a wide array of perturbations spanning from obesity, insulin resistance, type 2 diabetes, the metabolic syndrome, and cardiovascular disease risk and mortality in both adults and children [1]. Among these, excessive daytime sleepiness (EDS) is an undervalued public health issue that is often underdiagnosed and poorly addressed [2]. Variations in the definition of EDS and limitations in clinical assessment lead to difficulties in its epidemiological study, but the relevance of this symptom from a socioeconomic perspective is inarguable [2]. EDS often stems from sleep disturbances caused by psychological disorders, medications, environmental factors, poor sleep hygiene, age-related chronobiological changes, or underlying sleep disorders [2]. Of interest, growing evidence highlights a connection between EDS and type 2 diabetes. A recent hospital-based cross-sectional study conducted among 229 Ethiopian patients with type 2 diabetes reported a 27.1% prevalence of EDS [3]. The study identified poor glycemic control—defined as fasting blood glucose levels ≥ 126 mg/dL, assessed through three consecutive measurements—as a significant factor associated with EDS [3]. Similarly, other studies have demonstrated that individuals with type 2 diabetes who experience EDS, as measured by tools such as the Epworth Sleepiness Scale (ESS), are more likely to suffer from severe hypoglycemia [4,5]. These findings suggest that EDS is closely associated with metabolic regulation in type 2 diabetes, with a possible bidirectional relationship with poor glycemic control. Poor sleep quality, both short and long sleep duration, has been associated with poor glycemic control due to worsening of insulin resistance by sleep loss [6]. Conversely, in individuals with type 2 diabetes, when plasma glucose levels are very high, they manifest more episodes of nocturia (due to glucosuria) and consequent nighttime awakenings and sleep disturbances [7], which, in turn, can worsen EDS. A prior study in a small group of participants (n = 311) suggested that poor glycemic control was positively correlated with the level of daytime sleepiness (measured by ESS > 10) in normal weight men, but no such association was seen in men with obesity or women in any BMI category [8]. There is a knowledge gap with regard to this association—between EDS and glycemic control—in an adequately powered Latino population of Mexican ancestry, considering the burden of type 2 diabetes in this minority underrepresented population.

Latino individuals are disproportionately affected by both type 2 diabetes [9] and sleep disorders [10], placing them at a higher risk of related adverse health outcomes. In addition, sleep health disparities (SHDs) are defined as persistent differences in dimensions of sleep health—such as duration, efficiency, timing, regularity, alertness, and quality—and are particularly pronounced among Latino individuals when compared to non-Latino Whites [10]. These disparities, compounded by structural inequities, such as racial segregation, limited healthcare access, and environmental exposures, further contribute to the elevated burden of poor sleep health and associated health risks within Latino communities [10].

To address these disparities, El Banco por Salud (El Banco), a biobank established in 2018 by the Center for Disparities in Diabetes, Obesity, and Metabolism at the University of Arizona, serves as a critical resource [11]. It includes predominantly self-reported Latino individuals (>98%) of Mexican ancestry receiving care at Federally Qualified Health Centers (FQHCs) in southern Arizona, such as El Rio Community Health Center and Mariposa Community Health Center. El Banco provides a repository of comprehensive health data and biospecimens, facilitating research on diet-sensitive conditions like type 2 diabetes in this high-risk population [11].

Given the disproportionate burden of sleep disorders among Latino individuals and their potential impact on glycemic control and cardiometabolic health, this study aimed to (1) characterize daytime sleepiness status within the El Banco cohort and (2) investigate the association of EDS with cardiometabolic risk factors and glycemic control in this underserved population.

## 2. Materials and Methods

### 2.1. Study Sample

This cross-sectional study analyzed complete data from 1685 participants in the El Banco biobank. El Banco is an ongoing cohort study initiated in 2018, comprising predominantly self-identified Latino participants recruited through collaborative partnerships with FQHCs in southern Arizona. The cohort includes probands (index participants) and their biologically related or cohabiting family members, as well as close family friends who met eligibility criteria and provided informed consent [11]. Potential participants were prescreened via electronic health records based on the following criteria: (1) self-reported Latino ethnicity, (2) age 18–75 years, and (3) glycated hemoglobin (HbA1c) ≥ 39 mmol/mol (5.7%). Non-diabetic individuals were enrolled as family members or affiliates referred by probands. For the present investigation, we selected a subset of participants who met the following additional inclusion criteria: (1) availability of complete EDS assessment data and (2) all core demographic and metabolic parameters. Demographic and anthropometric measurements were obtained using standardized protocols, as previously described [11]. This study received ethical approval from the University of Arizona Institutional Review Board, and all participants provided written informed consent. Research data were managed using the University of Arizona’s REDCap (Research Electronic Data Capture) system, ensuring HIPAA-compliant data collection, quality control, and secure export [12].

### 2.2. Daytime Sleepiness Assessment

EDS is defined as an overwhelming need to sleep during normal waking hours, leading to frequent napping or falling asleep unintentionally [2]. This condition can significantly impact daily life and functioning. It is distinct from general fatigue, as it involves a more profound and persistent desire to sleep [2]. The Epworth Sleepiness Scale (ESS) is a widely used tool for assessing daytime sleepiness in research studies [13]. It evaluates the degree of subjective daytime sleepiness in eight common everyday situations. Each question is scored from 0 (no chance of dozing) to 3 (high chance of dozing), resulting in a total score ranging from 0 to 24. A score above 10 indicates EDS, while scores of 10 or below are classified as normal daytime sleepiness [8,13].

### 2.3. Physical Activity Assessment

Physical activity was quantified using the validated Godin–Shephard Leisure-Time Physical Activity Questionnaire (GSLTPAQ) [14]. The Leisure-Time Score (LTS) was computed as a weighted sum of self-reported weekly physical activity sessions, accounting for exercise intensity. Specifically, mild-intensity activities were assigned a metabolic equivalent (MET) coefficient of 3, moderate-intensity activities a coefficient of 5, and vigorous-intensity activities a coefficient of 9. The total LTS was calculated by multiplying the weekly frequency of each activity intensity by its respective MET value and summing the products, yielding a composite measure of leisure-time energy expenditure. Based on the LTS results, physical activity levels were categorized as active (LTS ≥ 24), moderately active (LTS = 18–24), or sedentary (LTS ≤ 18) [14].

### 2.4. Sociodemographic Characteristics

Sociodemographic data were obtained from standardized questionnaires completed by participants. The analyzed variables comprised participant type (proband versus family member), language spoken at home (categorized as Spanish-only, more Spanish than English, both equally, more English than Spanish, or English-only), educational level (less than a high school education or at least a high school diploma), employment status (stratified as full-time, part-time, or unemployed), marital status (classified as single, married or in a couple, widowed, divorced, or separated), country of birth (USA or non-USA), type of insurance (Medicare, commercial, Medicaid, none, or unknown), and annual household income. The mean annual household income was a census tract average obtained from zip codes utilizing the government site (census.gov), as previously reported [15].

### 2.5. Dietary Assessment

Dietary patterns were evaluated using the validated Brief Dietary Assessment Tool for Hispanics, a 23-item instrument specifically developed for Hispanic/Latino populations based on NHANES III data from Mexican Americans [16]. This culturally adapted tool quantifies daily consumption of fruits, vegetables, and dietary fats through two distinct subscales. The dietary fat subscale assesses consumption frequency of 16 common high-fat food items (e.g., French fries and fried potatoes) over the preceding month, with response options ranging from ≤1 time/month to ≥5 times/week. The fruit/vegetable subscale evaluates intake frequency of seven produce categories (e.g., leafy greens and citrus fruits) during the same period, with responses scaled from <1 time/week to ≥2 times/day. This instrument was selected for its demonstrated validity in capturing characteristic dietary patterns among Hispanic communities while maintaining practical administration in clinical and research settings [16].

Alcohol use behaviors were evaluated using the abbreviated Alcohol Use Disorders Identification Test-Consumption (AUDIT-C) questionnaire, a validated screening instrument for hazardous drinking patterns [17]. The AUDIT-C yields a composite score ranging from 0 to 12 points, where 0 indicates complete alcohol abstinence. Based on established clinical thresholds, participants were classified as at-risk drinkers if they exceeded gender-specific cutoff scores: >4 points for male individuals and >3 points for female individuals. These thresholds identify individuals with an increased likelihood of alcohol-related health consequences. The AUDIT-C demonstrates a dose-response relationship between total score and probability of alcohol-associated harm, with higher scores indicating greater risk for negative health outcomes and safety concerns [17].

### 2.6. Cardiometabolic Risk Factors

According to the Adult Treatment Panel III, cardiometabolic risk factors were defined as follows: dyslipidemia (triglycerides ≥ 150 mg/dL), elevated fasting blood glucose (≥5.6 mmol/L), high-density lipoproteins (HDL) cholesterol (<40 mg/dL for men and <50 mg/dL for women), hypertension (systolic blood pressure > 130 mmHg and diastolic > 85 mmHg), and large waist circumference (WC) (men ≥ 40 inches and women ≥ 35 inches) [18]. Each cardiometabolic risk factor was coded as either “1” for having the factor or “0” for not having the factor. All measurements were performed by trained study staff in a clinical laboratory setting.

### 2.7. Statistical Analysis

All analyses were performed using IBM SPSS Statistics (Version 21.0, SPSS Inc., Chicago, IL, USA). Continuous variables were expressed as mean ± standard deviation (SD), while categorical variables were summarized using absolute frequencies and percentages (n, %). Normality of distribution was verified through Kolmogorov–Smirnov testing; non-normally distributed variables underwent logarithmic transformation prior to parametric analysis. Initial analyses focused on generating descriptive statistics to characterize both the overall study population and subgroups stratified by EDS status. Comparative analyses between individuals with and without EDS were performed using independent samples t-tests for continuous variables and Pearson’s chi-square tests for categorical variables. Additionally, a stratified analysis by sex was conducted to explore potential sex-specific associations between EDS and cardiometabolic risk factors. This exploratory analysis aimed to evaluate whether sex differences may influence or drive some of the associations observed in the overall sample. The association between daytime sleepiness scores and HbA1c levels was examined through a series of linear regression models. HbA1c served as the continuous dependent variable, with daytime sleepiness score as the primary independent variable. Three sequential models were constructed: Model 1 represented the crude, unadjusted association. Model 2 incorporated adjustments for age, sex, body mass index (BMI), and waist WC. Model 3 extended these adjustments to include sociodemographic covariates (home language, employment status, marital status, country of birth, insurance status, and household income). A secondary analysis evaluated the relationship between dietary components and daytime sleepiness scores using linear regression, with adjustments for age, sex, BMI, and HbA1c levels. Model diagnostics included visual inspection of error residual histograms to verify normality assumptions and assessment of heteroscedasticity.

Statistical significance was defined as *p* < 0.05 for all analyses.

### 2.8. Statistical Power Calculation

Post-hoc power analysis was performed using GPower 3.1 (α = 0.05, N = 1685), converting the observed R^2^ values into effect sizes (f^2^) to compute statistical power. All models demonstrated excellent power (1-β > 0.999), including for the smallest effects (f^2^ = 0.011).

## 3. Results

Descriptive characteristics, cardiometabolic risk factors, and HbA1c of the entire study population and according to daytime sleepiness status are presented in Table 1.

The analyses included 1685 participants with a mean age of 52.6 ± 14.3 years. Of these, 1314 (78.0%) were classified as participants without daytime sleepiness and 371 (22.0%) as participants with EDS. Most participants were women (67.7%), with obesity (mean BMI 32.4 ± 7.0 kg/m^2^), and were sedentary (44.1%). Participants with EDS had a significantly higher BMI (*p* < 0.001) and a larger mean WC (*p* = 0.002) compared to participants without daytime sleepiness. Lifestyle assessment showed that participants with EDS were more sedentary compared to participants without daytime sleepiness (49.3 vs. 42.6%, *p* = 0.017). When stratified by sex, differences in BMI, WC, and physical activity remained significant in males, with participants with EDS having a higher BMI (33.4 ± 7.9 vs. 31.6 ± 6.9, *p* = 0.001) and WC (45.0 ± 7.1 vs. 43.2 ± 6.5, *p* = 0.007), as well as being more sedentary (45.9% vs. 34.0%, *p* = 0.012) compared to those without daytime sleepiness. In females, participants with EDS had a significantly higher BMI (33.7 ± 7.4 vs. 32.4 ± 6.7, *p* = 0.007), while no significant differences were observed for WC or physical activity.

Regarding cardiometabolic risk factors and HbA1c levels, participants with EDS had a higher prevalence of large WC (*p* = 0.003), dyslipidemia (*p* = 0.036), and significantly higher HbA1c levels (*p* = 0.007) compared to participants without daytime sleepiness. When stratified by sex, differences in dyslipidemia and HbA1c remained significant in females, with participants with EDS having a higher prevalence of dyslipidemia (51.4% vs. 42.7%, *p* = 0.015) and higher HbA1c levels [58.9 ± 23.4 mmol/mol (7.5 ± 2.1%) vs. 55.1 ± 21.7 mmol/mol (7.2 ± 2.0%), *p* = 0.012]. In males, only the prevalence of large WC remained significant, with participants with EDS having a higher prevalence of large WC compared to those without daytime sleepiness (79.3% vs. 70.2%, *p* = 0.047).

Table 2 presents the sociodemographic characteristics of the entire study population and stratified by daytime sleepiness status.

Table 3 shows the linear regression analyses examining the association between dietary components and daytime sleepiness, adjusted for age, sex, BMI, and HbA1c levels.

An increase in the daytime sleepiness score (more EDS) was significantly associated with an increase in the frequency of consumption of flour tortillas (*p* = 0.031), hamburgers/cheeseburgers (*p* = 0.004), any French fries/fried potatoes (*p* = 0.007), fried chicken (*p* = 0.014), tacos/burritos/enchiladas (*p* = 0.011), pizza (*p* = 0.034), roast pork, beef, or steak (*p* = 0.026), cake, sweet rolls, and doughnuts (*p* < 0.001), salad dressing (*p* = 0.005), potato/corn chips (*p* = 0.005), fruit juice (*p* < 0.001), fruit (fresh/frozen/canned) (*p* = 0.035), and any potatoes (*p* = 0.002) and soft drinks (*p* < 0.001).

Table 4 presents the results of linear regression analyses examining the association between daytime sleepiness score and HbA1c levels, with different models accounting for various adjustments.

Daytime sleepiness score was significantly associated with higher HbA1c levels in the unadjusted model (*p* < 0.001), and this relationship remained significant after adjusting for age, sex, BMI, and WC (*p* < 0.001). Even with further adjustments for additional sociodemographic factors, the positive association between daytime sleepiness score and HbA1c levels remained statistically significant (*p* = 0.004).

## 4. Discussion

This study presents the results of an analysis of a cohort of 1685 individuals who self-report as Latinos of Mexican ancestry. In this cohort, individuals with EDS (22.0%), as measured by the ESS, were more likely to exhibit higher BMI and larger WC compared to those without daytime sleepiness. This finding aligns with existing evidence linking poor sleep quality to adiposity, reflecting the well-documented bidirectional relationship between sleep disorders and obesity [19]. Although EDS is a symptom rather than a distinct sleep disorder, it can often result from underlying sleep disorders, such as obstructive sleep apnea, sleep disturbances, short or long sleep duration, or circadian rhythms, which are themselves associated with hormonal dysregulation—such as increased ghrelin and reduced leptin levels—that promote weight gain [20,21,22]. Conversely, obesity can exacerbate sleep disturbances and thereby further perpetuate this cycle [19,23]. Notably, evidence suggests that beyond sleep duration and quality, the timing of sleep may also play a role in metabolic risk. A study on Hispanic/Latino adults found that later sleep timing was associated with higher insulin resistance (HOMA-IR) and fasting plasma glucose levels, particularly among older participants, highlighting the potential metabolic consequences of disrupted circadian rhythms [22].

Our findings also revealed disparities in EDS prevalence based on sociodemographic factors. Individuals with EDS were more likely to have Medicare insurance compared to participants without daytime sleepiness, which may reflect an association with older age, increased comorbidities, or socioeconomic factors [24] that could influence access to healthcare and sleep interventions. This may partly reflect an association with older age, as Medicare is typically available to individuals aged 65 and older, who are more likely to experience comorbid conditions that contribute to sleep disturbances. While the majority of individuals with Medicare are over 65, it is important to acknowledge that younger individuals with disabilities may also qualify for Medicare or Medicaid based on income [24]. This subgroup may face compounded risks related to physical or mental health conditions, limited access to quality sleep care, and socioeconomic disadvantages, all of which may increase vulnerability to EDS [25]. The association between EDS and Medicare coverage could therefore serve as a proxy for increased health burden, functional limitations, or reduced access to preventive care and sleep interventions. In addition, these findings align with national data showing that Medicare beneficiaries with untreated insomnia have significantly higher health care utilization and costs across all points of care, especially for inpatient care [26]. This suggests that undiagnosed or unmanaged sleep disorders, such as insomnia or EDS, may contribute to higher health care resource utilization not only for sleep-related problems but also for other comorbid conditions. Thus, individuals with public insurance may represent a clinically and socioeconomically vulnerable subgroup whose unmet sleep health needs could exacerbate the broader health burden [26].

Participants with EDS reported higher frequencies of consuming calorie-dense, nutrient-poor foods, including flour tortillas, hamburgers/cheeseburgers, French fries, fried chicken, tacos/burritos/enchiladas, pizza, roast pork/beef/steak, cake/sweet rolls/doughnuts, salad dressing, potato/corn chips, fruit juice, fruit, potatoes, and soft drinks. This pattern suggests a preference for energy-dense foods that may exacerbate metabolic dysfunction [27]. Consistently, previous findings from the El Banco biobank revealed low adherence to dietary and physical activity guidelines, alongside a high prevalence of cardiometabolic risk factors [28]. Moreover, these findings align with prior research demonstrating that sleep deprivation and EDS increase cravings for high-calorie foods, likely driven by alterations in reward-related brain activity and hormonal imbalances [29]. In addition, this kind of eating pattern, high in processed foods, added sugars, and saturated fats and low in fruits, vegetables, and fiber, is often linked to a sedentary lifestyle [30]. In this regard, our results showed that participants with EDS were more sedentary compared to those without daytime sleepiness. The existing literature supports the bidirectional relationship between physical activity and sleep, suggesting that physical inactivity contributes to poor sleep quality, while poor sleep quality, in turn, results in decreased physical activity [31]. While unhealthy dietary preferences, as seen in this case, may not directly reduce physical activity, their well-known associations with obesity and related health issues, such as reduced mobility and increased fatigue [30], could discourage physical engagement [32]. Conversely, healthier dietary preferences, characterized by greater consumption of fruits, vegetables, whole grains, and lean proteins, may support physical activity by enhancing energy availability, improving overall health, and providing better nutritional balance [33]. Finally, regular physical activity may promote healthier eating habits, contributing to a positive feedback loop [34].

From a cardiometabolic perspective, EDS was significantly associated with a higher prevalence of dyslipidemia and elevated HbA1c levels. Notably, the association between EDS and HbA1c remained significant even after adjusting for variables such as age, sex, BMI, WC, and sociodemographic factors. Regarding glycemic control as measured by HbA1c, despite the highly significant association, the proportion of variance in HbA1c explained by the EDS score was low, at most about 10%, depending on the statistical model used. Beta coefficients for the analysis comparing HbA1c as a dependent variable suggest that a unit change in EDS was associated with at most a 0.068 to 0.103 change in HbA1c. This suggests a multifactorial basis of the regulation of glycemic control, and the results of this study established EDS and, by extension, sleep as one of these modifiable factors that could be considered as part of a multifaceted lifestyle intervention to improve glycemic control. While some evidence exists regarding the link between EDS and fasting glucose or hypoglycemic events in individuals with type 2 diabetes [3,4,5], to our knowledge, the data connecting EDS with glycemic control in a prior study indicated that this association was primarily seen in men without obesity, whereas it did not appear to be significant in men with obesity or in women, regardless of obesity status [8]. Moreover, there are no studies examining the connection between EDS and HbA1c in Latino individuals of Mexican ancestry, such as the one in our study. This highlights the novelty of our findings.

In fact, while the association between EDS and cardiometabolic risk factors has been documented in prior research, our study provides novel insights into this relationship specifically within an underserved Latino population of Mexican ancestry, a group disproportionately affected by type 2 diabetes yet underrepresented in sleep-health studies. Unlike previous work focused on general populations or clinical cohorts, our findings reveal that EDS is independently associated with poorer glycemic control (HbA1c) in this demographic, even after adjusting for sociodemographic and lifestyle confounders. This underscores the need for culturally tailored interventions targeting sleep health in Latino communities, where disparities in diabetes outcomes persist.

From a physiological standpoint, EDS may disrupt glycemic control through several mechanisms. Evidence links EDS to insulin resistance, independently of obesity, with studies showing associations between daytime sleepiness, fasting insulin levels, and HOMA-IR [35,36]. Sleep fragmentation further exacerbates insulin resistance via heightened sympathetic and adrenocortical activity [37].

Recent studies have demonstrated that GLP-1 receptor agonists can effectively reduce EDS in individuals with obesity and type 2 diabetes. Liraglutide treatment significantly decreased EDS after 1 and 3 months, accompanied by improvements in metabolic outcomes such as weight loss and better glycemic control [38]. Similarly, the SURMOUNT-OSA trial showed that Tirzepatide treatment led to significant reductions in daytime sleepiness, as measured by the PROMIS-SRI T-score, compared to placebo [39]. While GLP-1 receptor agonists could disrupt the bidirectional relationship between daytime sleepiness and glycemic control, limited access to these medications among Latino populations may exacerbate health disparities. Therefore, dietary, exercise, and sleep-based interventions should be strengthened alongside efforts to improve access to these therapies and address supply chain challenges.

This study has several strengths. These include, first, the use of a large, well-characterized cohort of Latino individuals of Mexican ancestry with type 2 diabetes, a population at high risk for both EDS and cardiometabolic complications. Second, the detailed assessment of dietary patterns, cardiometabolic risk factors, and sociodemographic characteristics allowed for a comprehensive analysis of the associations between EDS and health outcomes. Finally, the use of multivariable regression models accounted for key confounders, strengthening the validity of our findings.

However, this study also has limitations. The cross-sectional design precludes establishing causality between EDS and metabolic outcomes, as the observed relationships may be bidirectional. Additionally, as this analysis utilized existing biobank data, no a priori power calculation was performed; however, post-hoc analysis confirmed adequate power for primary outcomes. While key cardiometabolic parameters (HbA1c, lipids, and anthropometrics) were objectively measured, dietary, physical activity, and sleepiness data relied on self-report. Although validated instruments were used, potential reporting biases (e.g., recall error and social desirability) should be considered when interpreting these associations. Finally, we did not account for caffeine intake, the potential inclusion of night shift workers, or the presence of sleep disorders and medication use, all of which could influence the observed associations.

## 5. Conclusions

This study provides compelling evidence that EDS is significantly associated with obesity, poor dietary patterns, and impaired glycemic control in Latino individuals of Mexican ancestry at high risk for type 2 diabetes. Our findings make three key contributions to the field: First, we confirm that the well-established relationship between EDS and metabolic dysfunction extends to this understudied population. Second, we identify population-specific associations between EDS and unhealthy eating behaviors that may help explain the observed glycemic dysregulation. Third, we demonstrate that the EDS-HbA1c relationship persists even after accounting for traditional risk factors, suggesting sleep health should be considered in diabetes management strategies for this community. As the first large-scale investigation of these relationships in this high-risk population, our work fills an important knowledge gap while reinforcing the universality of sleep-metabolism connections. These findings highlight the need for culturally tailored interventions that address both sleep health and dietary behaviors in Latino communities. Future research should examine whether targeted management of EDS can improve glycemic outcomes beyond standard diabetes care.

## Figures and Tables

**Table 1:** Descriptive characteristics, cardiometabolic risk factors, and HbA1c of the entire study population and according to daytime sleepiness status.

Parameters	N of Respondents	All	Without Daytime Sleepiness (ESS ≤ 10) N = 1314, 78.0%	With EDS (ESS > 10) N = 371, 22.0%	*p* Value
**Age (years)**	1685	52.6 ± 14.3	52.5 ± 14.5	52.8 ± 13.8	0.747
**Sex**	1684				
Male (N, %)		544, 32.3%	421, 32.1%	123, 33.2%	0.612
Female (N, %)		1140, 67.7%	892, 67.9%	248, 66.8%	
**BMI (kg/m^2^)**	1676	32.4 ± 7.0	32.1 ± 6.8	33.6 ± 7.6	**<0.001**
**WC (inch)**	1670	42.1 ± 6.3	41.8 ± 6.3	43.0 ± 6.5	**0.002**
**WC (cm)**	1670	106.9 ± 16.0	106.2 ± 16.0	109.2 ± 16.5	**0.002**
**Physical activity**	1666				
Sedentary (n, %)		734, 44.1%	552, 42.6%	182, 49.3%	**0.017**
Moderately active (n, %)		280, 16.8%	214, 16.5%	66, 17.9%	
Active (n, %)		652, 39.1%	531, 40.9%	121, 32.8%	
**Alcohol use**	1685				
At-risk drinking (n, %)		381, 22.6%	291, 22.1%	90, 24.3%	0.390
Not at-risk drinking (n, %)		1304, 77.4%	1023, 77.9%	281, 75.7%	
**Cardiometabolic risk factors**					
Large WC (yes) (n, %)	1669	1391, 82.6%	1074, 82.3%	317, 87.1%	**0.030**
Dyslipidemia (yes) (n, %)	1675	757, 44.9%	573, 43.8%	184, 50.0%	**0.036**
Elevated FPG (yes) (n, %)	1576	1046, 62.5%	801, 61.4%	245, 66.0%	0.102
Low HDL (yes) (n, %)	1643	918, 54.5%	707, 55.2%	211, 58.3%	0.295
Hypertension (yes) (n, %)	1665	528, 31.3%	407, 31.3%	121, 33.1%	0.530
**HbA1c (mmol/mol)**	1685	56.7 ± 22.8	55.3 ± 22.6	59.3 ± 23.3	**0.007**
**HbA1c (%)**	1685	7.3 ± 2.1	7.3 ± 2.1	7.6 ± 2.1	**0.007**

Data are expressed as means ± SD or number, percentage. Values in bold indicate significant differences. ESS, Epworth Sleepiness Scale; EDS, excessive daytime sleepiness; BMI, body mass index; WC, waist circumference; FPG, fasting plasma glucose; HDL, high-density lipoprotein; HbA1c, hemoglobin A1c.

**Table 2:** Sociodemographic characteristics of the entire study population and according to daytime sleepiness status.

Parameters	N of Respondents	All	Without Daytime Sleepiness (ESS ≤ 10) N = 1314, 78.0%	With EDS (ESS > 10) N = 371, 22.0%	*p* Value
**Participant type**	1685				
Proband (N, %)		933, 55.4%	690, 52.5%	243, 65.5%	**<0.001**
Family member/ Proband Family Friend (N, %)		752, 44.6%	624, 47.5%	128, 34.5%	
**Home language**	1679				
Only Spanish (N, %)		577, 34.4%	442, 33.7%	135, 36.6%	0.081
More Spanish than English (N, %)		387, 23.0%	318, 24.3%	68, 18.7%	
Both equally (N, %)		301, 17.9%	237, 18.1%	64, 17.3%	
More English than Spanish (N, %)		279, 16.6%	205, 15.6%	74, 20.1%	
Only English (N, %)		135, 8.0%	108, 8.2%	27, 7.3%	
**Education**	1679				
<High school (N, %)		47.5, 47.7%	614, 46.9%	187, 50.4%	0.239
≥High school (N, %)		878, 52.3%	694, 53.1%	184, 49.6%	
**Work**	1657				
Full-time (N, %)		493, 29.8%	392, 30.3%	101, 27.7%	0.103
Part-time (N, %)		218, 13.2%	179, 13.9%	39, 10.7%	
Unemployed (N, %)		946, 57.1%	721, 55.8%	225, 61.6%	
**Marital status**	1684				
Single, never married (N, %)		363, 21.6%	275, 20.9%	88, 23.7%	0.304
Married or domestic partnership (N, %)		844, 50.1%	676, 51.5%	168, 45.3%	
Widowed (N, %)		121, 7.2%	94, 7.2%	27, 7.3%	
Divorced (N, %)		267, 15.9%	200, 15.2%	67, 18.1%	
Separated (N, %)		89, 5.3%	68, 5.2%	21, 5.7%	
**Country of birth**	1668				
Not US (N, %)		975, 58.5%	768, 59.0%	207, 56.4%	0.367
US (N, %)		693, 41.5%	533, 41.0%	160, 43.6%	
**Insurance**	1683				
Medicare (N, %)		547, 32.5%	404, 30.8%	143, 38.6%	**0.003**
Commercial (N, %)		272, 16.2%	212, 16.1%	60, 16.2%	
Medicaid (N, %)		328, 19.5%	251, 19.1%	77, 20.8%	
None (N, %)		310, 18.4%	264, 20.1%	46, 12.4%	
Unknown (N, %)		226, 13.4%	182, 13.9%	44, 11.9%	
**Household income (USD/year)**	1672	53,082.0 ± 12,155.1	52,971.5 ± 11,885.2	53,470.7 ± 13,070.0	0.486

Data are expressed as means ± SD or number, percentage. EDS, excessive daytime sleepiness. The mean household income was a census tract average obtained from zip codes utilizing the government site (census.gov). ESS, Epworth Sleepiness Scale; EDS, excessive daytime sleepiness; US, United States; USD, United States Dollar. Significant differences were observed in participant type and insurance (in bold). Specifically, participants with EDS were more likely to have Medicare coverage (*p* = 0.003). No other significant differences were observed.

**Table 3:** Linear regression analysis with dietary components of the Brief Dietary Assessment Tool for Hispanics questionnaire as dependent variables (outcomes) and Epworth Sleepiness Scale score as independent variable (predictor).

Outcomes	R^2^	Beta	CI (95%)	*p* Value	Overall *p* Value
**Dietary fat sources (times/month)**					
Flour tortilla	0.017	0.053	0.01–0.04	0.031	<0.001
Refried beans	0.017	0.043	−0.01–0.03	NS	<0.001
Hamburgers/cheeseburgers	0.103	0.069	0.01–0.03	0.004	<0.001
French fries/fried potatoes	0.072	0.065	0.01–0.03	0.007	<0.001
Fried chicken	0.013	0.061	0.01–0.03	0.014	<0.001
Eggs	0.005	0.021	−0.01–0.03	NS	0.112
Tacos/burritos/enchiladas	0.047	0.062	0.01–0.03	0.011	<0.001
Other mixed dishes with meat	0.050	0.039	−0.01–0.03	NS	<0.001
Pizza	0.129	0.049	0.01–0.02	0.034	<0.001
Roast pork, beef, or steak	0.036	0.054	0.01–0.03	0.026	<0.001
Cheese/cheese spread	0.008	0.040	−0.01–0.03	NS	0.016
Cake, sweet rolls, doughnuts	0.012	0.091	0.01–0.04	<0.001	0.002
Use fat or oil to fry, cook, or season	0.007	0.038	−0.01–0.03	NS	0.034
Salad dressing	0.026	0.069	0.01–0.03	0.005	<0.001
Potato/corn chips, peanuts	0.072	0.067	0.01–0.03	0.005	<0.001
Whole milk	0.008	0.022	−0.01–0.03	NS	0.022
**Dietary fruit and vegetable sources (times/week)**					
Green salad	0.028	0.014	−0.02–0.03	NS	<0.001
Fresh vegetables	0.018	−0.040	−0.05–0.01	NS	<0.001
Fruit juice	0.025	0.079	0.03–0.09	<0.001	<0.001
Fruit (fresh/frozen/canned)	0.008	0.052	0.01–0.08	0.035	0.029
Any potatoes	0.027	0.076	0.01–0.04	0.002	<0.001
Tomatoes/fresh salsa	0.017	0.018	−0.02–0.04	NS	<0.001
Vegetable stew/soup	0.004	0.019	−0.01–0.03	NS	0.191

Age, sex, BMI, and HbA1c were included as covariates in the model. CI, confidence interval; NS, not significant.

**Table 4:** Linear regression analysis with HbA1c (mmol/mol) as dependent variable (outcome) and Epworth Sleepiness Scale score as independent variable (predictor).

Outcome (HbA1c, mmol/mol)	R^2^	Beta	CI (95%)	*p* Value	Overall *p* Value
Model 1 ^a^					
**ESS score**	0.011	0.103	0.001–0.003	**<0.001**	<0.001
Model 2 ^b^					
**ESS score**	0.088	0.088	0.001–0.003	**<0.001**	<0.001
Model 3 ^c^					
**ESS score**	0.107	0.068	0.001–0.003	**0.004**	<0.001

^a^ unadjusted. ^b^ adjusted for age, sex, BMI, and WC. ^c^ adjusted for age, sex, BMI, and WC, home language, work, marital status, education, country of birth, insurance, and household income. Values in bold indicate significant differences. HbA1c, hemoglobin A1c; ESS, Epworth Sleepiness Scale; CI, confidence interval.

## Data Availability

The datasets generated during and/or analyzed in the current study are available from the corresponding author upon reasonable request.

## Abbreviations

The following abbreviations are used in this manuscript: AUDIT-C: Alcohol Use Disorders Identification Test—Concise version, BMI: Body Mass Index, CI: Confidence Interval, EDS: Excessive Daytime Sleepiness, ESS: Epworth Sleepiness Scale, FPG: Fasting Plasma Glucose, FQHC: Federally Qualified Health Centers, GLP-1: Glucagon-Like Peptide-1, HbA1c: Glycated Hemoglobin, HDL: High-Density Lipoprotein, HOMA-IR: Homeostasis Model Assessment of Insulin Resistance, LTS: Leisure-Time Score, SD: Standard Deviation, SHD: Sleep Health Disparities, WC: Waist Circumference.
